# DNA Damage and Deficiencies in the Mechanisms of Its Repair: Implications in the Pathogenesis of Systemic Lupus Erythematosus

**DOI:** 10.1155/2018/8214379

**Published:** 2018-07-12

**Authors:** Martha Paola Mireles-Canales, Susana Aideé González-Chávez, Celia Maria Quiñonez-Flores, Ever Adán León-López, César Pacheco-Tena

**Affiliations:** Facultad de Medicina y Ciencias Biomédicas, Universidad Autónoma de Chihuahua, Chihuahua, CHIH, Mexico

## Abstract

Systemic lupus erythematosus (SLE) is a perplexing and potentially severe disease, the pathogenesis of which is yet to be understood. SLE is considered to be a multifactorial disease, in which genetic factors, immune dysregulation, and environmental factors, such as ultraviolet radiation, are involved. Recently, the description of novel genes conferring susceptibility to develop SLE even in their own (monogenic lupus) has raised the interest in DNA dynamics since many of these genes are linked to DNA repair. Damage to DNA induces an inflammatory response and eventually triggers an immune response, including those targeting self-antigens. We review the evidence that indicates that patients with SLE present higher levels of DNA damage than normal subjects do and that several proteins involved in the preservation of the genomic stability show polymorphisms, some of which increase the risk for SLE development. Also, the experience from animal models reinforces the connection between DNA damage and defective repair in the development of SLE-like disease including characteristic features such as anti-DNA antibodies and nephritis. Defining the role of DNA damage response in SLE pathogenesis might be strategic in the quest for novel therapies.

## 1. Introduction

Systemic lupus erythematosus (SLE) is a heterogeneous and complex autoimmune disease; it is associated with the production of autoantibodies and inflammatory damage of multiple organs. The pathogenesis of SLE is not completely understood and is considered to be a multifactorial disease. It involves genetic factors and environmental factors; amongst the latter, ultraviolet radiation (UV) is consistently recognized as an activating and worsening factor [1]; its direct and initial effects are detectable in the skin of SLE patients.

Lupic dermatitis is frequently noted at the earliest stages of the disease and affects 75% of SLE patients along the course of the disease [2]. Skin involvement is important in the detection of SLE patients. Such importance is evidenced in the structure of the new SLICC (Systemic Lupus International Collaborating Clinics) criteria set for the classification of patients with SLE, which has expanded the catalog of skin manifestations as criteria to ease the classification of otherwise nonclassifiable SLE patients. The current version includes several subsets of cutaneous lupus erythematosus (CLE): acute (ACLE) (bullous SLE, toxic epidermal necrolysis), subacute (SCLE), chronic cutaneous lupus (CCLE) (discoid lupus, lupus panniculitis, lupus erythematosus tumidus, and lupus chilblain), presence of oral or nasal ulcers, and noncicatricial alopecia [3]. It is evident that skin involvement in SLE patients represents a hallmark of the disease for most patients and is also an opportunity to understand some aspects of its pathogenesis. Notably, photosensitive lupic dermatitis specially provides a scenario to explore the relationship between UV radiation and its consequences in cell physiology, like the link between UV-induced DNA damage and its potential link to subsequent inflammation and immune activation [4, 5].

As mentioned, the etiology of lupus remains elusive; however, recent evidence increasingly suggests a subnormal detection of DNA damage and also impaired repairing could play a role in its pathogenesis. The UV is a known threat to DNA. Under physiological conditions, the keratinocyte is adapted to maintain genomic integrity despite UV, whereas in pathological conditions, if the responsible mechanisms are deficient, accumulated DNA damage leads to early cellular senescence or apoptosis. DNA damage triggers an array of cellular signaling pathways that sense, signal, and repair DNA lesions; this response is termed DNA damage response (DDR), and aside from optimizing the genome preservation, under stressful conditions, it does induce an inflammatory or immune responses [6–9]. DDR has been explored as a potential explanation in several pathogenic processes including carcinogenesis [10–12] and also in autoimmunity [5, 13–15].

The present review aims at combining and analyzing the experimental findings that postulate DNA damage as well as the deficiencies in the mechanisms of response to this damage as relevant factors involved in the pathogenesis of SLE.

## 2. DNA Damage by UV Radiation

It is now well known that solar radiation is genotoxic, with UV being the most mutagenic component [16]. UV light is defined as the region of the electromagnetic spectrum with wavelengths of 200 to 400 nm. The UV spectrum is divided into three categories: UV-A (315–400 nm), UV-B (280–315 nm), and UV-C (200–280 nm). The stratospheric layer of the Earth absorbs most of UV-B and the radiation below 295 nm. For this reason, UV-C's effect on humans is not important although it has the greater potential to damage biological structures [17–21]. UV light is one of the powerful agents that can induce a variety of mutagenic and cytotoxic DNA lesions, such as cyclobutane pyrimidine dimers (CPDs), 6-4 photoproducts (6-4PPs), and their Dewar valence isomers as well as DNA strand breaks (most of them single-strand breaks) by interfering the genome integrity [22].

UV induces biological damage through two different mechanisms. First, photons are directly absorbed by different cellular components (especially DNA and proteins) and that can lead to photoinduced reactions. Second are mechanisms of photosensitivity processes, where endogenous or exogenous sensitizers absorb UV indirectly. The electronically excited sensitizer can return to its harmless basal state through intramolecular disintegration processes or may damage different cellular components [19, 21]. Cell damage through the excitation of a photosensitizer can be caused by two types of pathways, which are dependent on their chemical properties. Type I sensitizers oxidize a target by removing an electron to generate a radical cation while type II sensitizers transfer energy to oxygen to produce singlet oxygen or ultimately other ROS [19, 21].

The DNA damage induced by UV depends on the wavelength of the photons that affect the cell. For UV-B, the direct absorption of light by DNA results in the dimerization reaction in which two pyrimidine bases (CC, CT and TT, TC) are juxtaposed, producing various types of lesions, mainly CPDs and 6-4 PPs [23–26]. This reaction occurs predominantly at sites containing a thymine, with TC and TT the most photoreactive. The resulting photoproduct creates a lesion that distorts the DNA helix, creating adducts that can stop the transcription and replication [27]. While the distortion of the helix created by 6-4 PPs is greater, CPDs are eliminated slowly and are responsible for 80% of the mutations produced by UV-B [28]. Other types of damage are single- or double-strand DNA breaks and modifications in bases such as 8-oxoguanine (8-oxoG), thymine glycol, photoproducts of 5-6-dihydrothymine, and cytosine photohydrates [29].

UV-A is poorly absorbed by DNA, and its genotoxic effects have been explained by the indirect action of ROS [23, 24]. ROS oxidatively modifies DNA to produce DNA base products such as 8-oxoG and thymine glycol and may even lead to the oxidation of 2′-deoxyguanosine 5′-triphosphate to produce 8-oxo-7,8-dihydro-2′-deoxyguanosine 5′-triphosphate which may be misincorporated into DNA [26]. Additionally, recent studies have shown that exposure to UV-A causes CPDs in a direct [29] and indirect [23, 24, 30, 31] way.

Although UV-B induces the production of more CPDs than UV-A at equimutagenic doses, UV-A-induced photoproducts are more mutagenic than those induced by UV-B [32]. The activation of antimutagenic responses, particularly the cell cycle control points (intra-S, G1/S, and G2/M) mediated by p53 and p95, has been reported to diminish in cells with lesions produced by UV-A. This can lead to replication of damaged DNA and accumulation of mutations [32].

## 3. UV Radiation and SLE

Numerous studies suggest that damage to DNA, by either UV, reactive oxygen species (ROS), or others, is a factor involved in the development of SLE [33–35]. DNA damage leads to accumulation of mutations, genomic instability, and cell death by apoptosis. The accumulation of apoptotic cells results in an excessive presentation of autoantigens and production of autoantibodies. Under normal conditions, UV-damaged DNA is sensed and repaired by the activation of complex multiprotein pathways, whose function is to maintain the integrity of DNA, adequate genome functionality, and cellular homeostasis [36]. The fact that several proteins are involved in specific roles in multistep processes widens the chances for dysfunction; besides, many of the proteins involved in genome stability have functional influencing polymorphisms. Therefore, individual resilience against DNA aggressors despite being critical for survival exhibits individual heterogeneity.

The skin is recognized as a target tissue in SLE. It is assumed that the skin plays a crucial role in the onset and perpetuation of lupus disease activity, and this inflammatory process is connected to the damage induced by UV. Exposure to UV has been confirmed as a worsening factor in SLE patients. It has been shown that UV can increase the activity of systemic disease and exacerbate preexisting skin lesions in about 90% of patients [37].

UV-A composes 90–95% of the solar radiation that reaches humans. This radiation penetrates the dermis and epidermis and can cause an increase in pigmentation by the induction of melanin. An intense and prolonged exposure can cause premature skin aging and oxidative processes. By contrast, UV-B is a minimum constituent of the solar radiation although it is the most active radiation of terrestrial sunlight. UV-B penetrates deep into the basal membrane of the epidermis, and it induces a significant reduction of antioxidants, thus affecting the protection of the skin against free radicals which are generated after exposure to terrestrial sunlight [20]. Free radical effects have been related to skin cancer, premature skin aging, development of inflammatory erythema, and possibly inflammatory diseases such as SLE [38, 39].

The processes explaining the onset of skin lesions in patients with lupus are not clear. However, it has been shown that antibodies, some of them anti-DNA, locate and bind in the dermoepidermal junction [40]. Moreover, lymphocytes infiltrate the perivascular spaces and also the stromal-epithelial junction of hair follicles and sweat glands [41, 42] suggesting a local chemoattracting environment.

In addition to anti-DNA antibodies, also anti-SSA/Ro are linked to the inflammation induced by UV in SLE patients [43]; that is, photosensitive subtypes such as SCLE are associated to the presence of anti-Ro. It has been found that UV-B-irradiated keratinocytes express nuclear and cytoplasmic antigens (SSA/Ro, RNP, and Sm) in the surface of the cell membrane [44–48]; this externalization might play a role in the exacerbation of skin symptoms. Cell redistribution of Ro60 and La caused by heat or UV radiation, and its interaction with cytoskeleton, is associated to HSP70 suggesting a connection to cellular stress [49].

Aside from autoantibodies, proteins linked to DDR might also participate in the inflammatory response. Interferon-gamma-inducible protein 16 (IFI16) is considered as a cytosolic sensor for double-stranded DNA [50], and in the case of DNA double-strand breaks, it binds to different proteins enhancing ATM-p53 signaling. IFI16 has been implicated in the etiopathogenesis of systemic autoimmune diseases due to its pleiotropic effect on the immune system [51, 52] and also in senescence and cancer [53]. IFI16, which is normally a nuclear protein, translocates to the cytoplasm in skin explant cells damaged by exposure to UV-B and also in cells from the skin lesion from SLE patients, and it has been found in supernatants, opening its potential as an intercellular mediator as well [54]. Patients with SLE and systemic sclerosis have higher titers of serum antibodies against IFI16 if compared with healthy individuals. In the specific case of SLE, anti-IFI16 antibodies had an inverse correlation to proteinuria and C3 hypocomplementaemia suggesting that actual actions of the unblocked IFI16 could play a role in nephritis [55].

UV, especially UV-B, is a potent inducer of apoptosis [56, 57]. It has been shown that the rate of apoptosis, local levels of proinflammatory cytokines, and translocation of autoantigens from the nucleus to the cell membrane correlate in a dose-dependent manner under UV. Low doses of UV-B induce caspase-dependent apoptosis and increase the presence of Sm, Ku, and DNA antigens in the nucleus of irradiated cells. Intermediate doses of UV-B alter apoptosis, increase in the levels of interleukin- (IL-) 1, and translocation of nuclear autoantigens towards the cell membrane. Meanwhile, high doses of UV-B induce cellular necrosis, and nuclear or cytosolic autoantigens are then released into the extracellular space [58]. Also, apoptotic cells have been shown to accumulate in the skin of CLE patients due to an impairment in their clearance [57].

In murine SLE models, exposure to UV-A exacerbates disease activity. In (NZBxNZW)F1, MRL/lpr, and BXSB, and in healthy murine models (BALB-C), UV-A results in a significant increment of the anti-double-stranded DNA levels, increased splenic B cell activity, glomerular inflammatory changes, and premature death in SLE models [59]. As can be noted, the impact of UV in SLE murine models does not limit to the skin but enhances serologic and clinical features including nephritis. It can be assumed that the changes induced by UV in the skin increase the release of proinflammatory mediators, which have a systemic consequence. We have explored potential explanations previously [36].

Exposure to UV also increases DNA antigenicity, and the autoantibodies from patients with SLE interact preferably with irradiated DNA and other nuclear antigens released from damaged skin cells [60] and develop cytotoxicity which is antibody-dependent [47].

## 4. DNA Damage in SLE

Native DNA is a poor immunogen, and there is a physiological rationale behind that; yet, anti-DNA antibodies in SLE patients are a hallmark of the disease and evidence the loss of the tolerance to self-DNA [61]. Although the origin of these autoantibodies is unknown, it has been shown that SLE patients have increased DNA damage (Table 1) as well as defects in the maintenance of genome stability and repair mechanisms. Also, anti-DNAs have higher affinity to DNA modified by oxidative stress [62–65], suggesting that if damaged DNA levels increase, it may play a role in the exacerbation of SLE.

There is evidence that the blood leukocytes of SLE patients show greater DNA damage than those of healthy controls do. In newly isolated and cultured neutrophils of SLE patients, nuclear DNA damage has been found to be significantly higher compared to patients with rheumatoid arthritis (RA) or healthy subjects [66]. In T-cells from SLE patients, damaged DNA, specifically single-strand DNA breaks, is higher than in healthy controls [67]. Likewise, an increase in DNA damage levels by double-stranded DNA break has been found in peripheral lymphocytes in SLE patients with high levels of anti-La/SSB and anti-RNP antibodies compared to SLE patients without these antibodies [68] suggesting a pathogenic role. A potential explanation for this damage could rely on a prooxidant/antioxidant imbalance which increases the plasma concentrations of malondialdehyde, a marker of oxidative stress, as well as by the decrease in the activity of the superoxide dismutase enzyme in these patients [69].

Even the damage to mitochondrial DNA (mtDNA) has been observed in PBMC of SLE patients compared to healthy individuals, and it has been observed that there is a correlation between high levels of damage to mtDNA with increased organ involvement [70].

The association of DNA damage with the pathogenesis of lupus has also been studied in *in vivo* models. Gehrke et al. repeatedly injected DNA damaged by UV-B into the earlobes of MRL/lpr mice. Immunostaining analysis showed the typical characteristics of cutaneous lesions found in SLE, including epidermotrophic inflammatory lymphocytic infiltrate and hydropic degeneration of the basement membrane and colloid bodies. Also, they increased the production of interferon I and double-stranded anti-DNA (anti-dsDNA) antibodies in response to UV-damaged DNA intravenous injection but not with undamaged DNA injection [5].

The experimental evidence described above shows the association between increased levels of DNA damage and the presence of SLE. The increase in DNA damage has been demonstrated in different cell types and *in vivo* systems, as well as in nuclei and mtDNA.

## 5. Deficiency in DNA Damage Response in SLE

**Figure 1:**
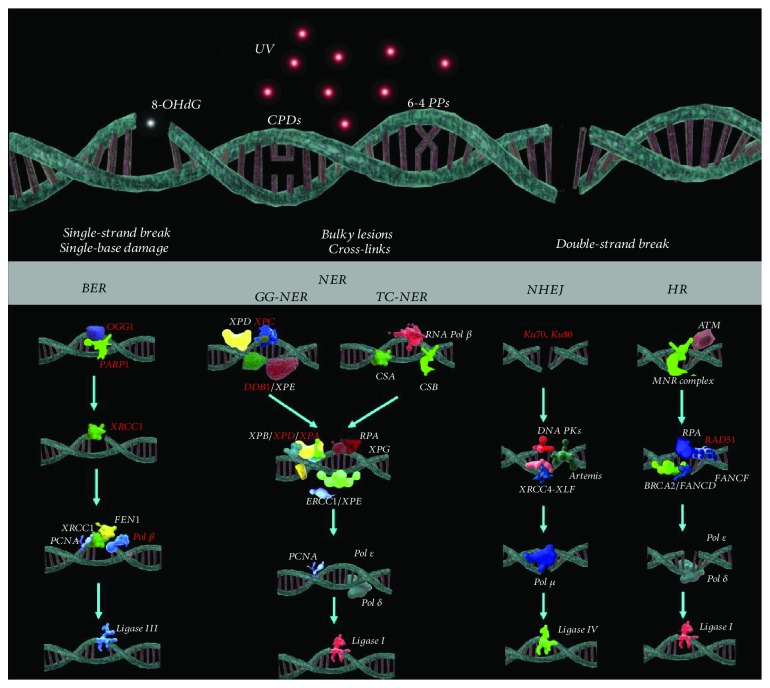
Enzymatic deficiencies in DNA repair pathways reported in systemic lupus erythematosus. The figure shows the main proteins involved in DNA repair mechanisms and highlights in red those enzymes that have been reported abnormal in SLE. Base excision repair (BER): repair of single-strand breaks and single-base damage (e.g., 8-oxodG). Nucleotide excision repair (NER): repair of bulky lesions and cross-links (e.g., CPDs and 6-4 PPs induced by UV). Nonhomologous end-joining (NHEJ): repair of double-strand breaks. Homologous recombination (HR): repair of double-strand breaks. SLE: systemic lupus erythematosus; DNA: deoxyribonucleic acid; 8-oxodG: 8-hydroxy-2′-deoxyguanosine; hOGG1: 8-oxoguanine DNA glycosylase; PARP: poly-ADP ribose polymerase; XRCC1: X-ray repair cross-complementing protein 1; ANAs: antinuclear antibodies: Pol *β*: polymerase beta; CPDs: pyrimidine cyclobutane dimers; 6-4 PPs: 6-4-pyrimidine pyrimidone photoproducts; UV: ultraviolet radiation; XPA: xeroderma pigmentosum complementation group A; XPC: xeroderma pigmentosum complementation group C; XPE: Xeroderma pigmentosum complementation group E; DDB1 DNA damage-binding protein 1.

The DNA of each of human cells undergoes at least 10^4^ lesions daily [71]; because of this, cells rely on specialized detection and repair proteins that scan the genome continuously for damage. Excision repair, which includes base excision repair (BER) and nucleotide excision repair (NER), is a complex multistep pathway, where the damaged DNA is replaced with a new one and plays an important role in DNA repair with the help of a number of glycosylases and polymerases, respectively (Figure 1) [72].

The BER corrects DNA damage from oxidation, deamination, and alkylation that cause little distortion to the DNA helix structure. In BER, an apurinic/apyrimidinic (AP) site is recognized by an AP endonuclease that introduces a nick immediately 5′ to the AP site, followed by repair synthesis, removal of the AP site, and finally ligation. The base release is catalyzed by one of at least 11 distinct DNA glycosylases that are specific for a particular set of lesions [73, 74].

The NER is the main mechanism to remove the mutagenic lesions 6-4 PPs and CPDs induced by UV in humans [75], and there are 20–30 proteins involved in this repair process that act in an established sequential order [76]. The NER process is subdivided into two mechanisms: first, global genome repair (GGR), which repairs localized lesions in the genome, and second, transcription-coupled repair (TCR) which repairs lesions in strands with active genes during transcription [77]. These two mechanisms differentiate only by the proteins involved in the initial detection of the DNA lesion. The TCR is initiated by the Cockayne syndrome A and B proteins (CSB, CSA) that regulate the recruitment of repair factors to the injury site and chromatin remodeling, whereas RNA polymerase II has to be temporarily removed from the DNA strand to allow its repair and subsequently restart transcription [78].

GGR is a process which detects DNA sequence damage by XPC proteins (xeroderma pigmentosum complementation group C) and XPE (xeroderma pigmentosum complementation group E) [79, 80]. XPC acts in a complex with hRAD23B and core 2. For some types of DNA lesions, including CPDs and 6-4 PPs, damage recognition is supported by proteins DDB1 and 2 (DNA damage-binding proteins 1 and 2). DDB proteins contribute to guiding the XPC-hRAD23B complex to the CPD/6-4 PP site [81].

Once the damaged DNA is detected either way, the two pathways converge to a common downstream pathway. The double DNA helix around the injury is unwound by the helicase activity of XPB and XPD which are components of the transcription factor IIH (TFIIH) [76]. The XPA and RPA proteins (replication protein A) determine the site of the cut. The XPF and XPG proteins are two endonucleases that act in a defined sequence to cut the DNA damaged strand. The XPG endonuclease cleaves the DNA strand on the 3′ side at about five nucleotides away from the lesion, and the ERCC1-XPF complex cuts the strand from the 5′ side [76, 82], thereby removing a strand section of 25–30 nucleotides. The gap generated by the resection is filled by DNA synthesis using *δ*/*ε* polymerase, and the new strand is sealed by DNA ligase [75, 76, 81, 83–85]. The consequences of NER defects can be explained by three autosomal recessive syndromes: xeroderma pigmentosum, Cockayne syndrome, and trichothiodystrophy [76].

In SLE patients, it has been observed that the NER DNA repair process is less efficient than in healthy individuals (Figure 1), and furthermore, this deficiency is even worse in patients with active nephritis, suggesting a pathogenic connection between the seriousness of the defective DNA repair and the autoimmune severity; such connection is consistent to that found in several murine models. Additionally, a negative regulation of the genes encoding the proteins involved in the NER pathway in SLE patients, specifically DDB1, ERCC2, XPA, and XPC, has been found [71, 86].

Deficiencies in the NER process are not the only defects in the DDR reported in individuals with SLE (Table 2). In numerous experimental studies, it has been detected that lymphocytes and neutrophils in SLE patients are less efficient in DNA repair [66, 71, 87]. It has also been found that in lymphoblastoid cells of patients with SLE, there is a deficiency in DNA repair mechanisms due to double-strand DNA breaks [87].

Senejani et al. created a murine model in which the POLB gene encoded Pol *β* (polymerase beta) with little activity. Pol *β* is a key enzyme in the DNA base excision repair (BER) pathway, which is necessary for DNA maintenance, replication, and recombination. The mouse that expressed this hypomorphic POLB allele developed pathological features similar to those present in SLE compared to the wild-type healthy murine model. These features include increased levels of immune complexes in glomeruli, elevated levels of serum antinuclear antibodies (ANAs), dermatitis, glomerulonephritis, and cervical lymphadenopathy with T and B lymphocyte infiltrates (Figure 1) [88].

DNase I is the nuclease most commonly found in serum, urine, and secretions. This nuclease has been linked to the removal of DNA from nuclear antigens at sites with high cell replication likely reducing its immunogenicity and under normal conditions reduces the likelihood of an autoimmune response. Decreased levels of DNase I have been found in SLE patients compared to healthy individuals. Möröy et al. created a DNase I-deficient mouse model. Mice exhibited the classic symptoms of SLE as well as some lab abnormalities like increased levels of ANAs. In the homozygous DNase I-deficient mice, high anti-DNA titers and also glomerulonephritis were present aside. This increased severity of both the clinical and serologic abnormalities in the homozygous mice correlates with the severity of the defective DNA processing with the seriousness of the disease abnormalities.

Also, several polymorphisms of the XRCC1 (X-ray cross-complement 1) protein, an important mediator in the BER pathway of DNA damaged by oxygen, ionization, and alkylating agents, are associated with SLE (Figure 1) [90, 91]. There are more than 300 single-nucleotide polymorphisms reported for XRCC1, of which the most common are Arg194Trp, Arg280His, and Arg399Gln. A decrease in the Arg/Gln genotype of the Arg399Gln polymorphism in SLE patients compared to healthy controls in the Iranian population has been reported [33], suggesting a potential protective role. Polymorphisms on Arg399Gln have also been described in relation to SLE in Taiwanese Han Chinese and Polish patients [90, 92]. A recent meta-analysis suggests a paradoxic effect of this polymorphism in Caucasian and Oriental populations [93] and did not show an association with rheumatoid arthritis susceptibility.

A high-sensitivity marker for DNA damage by ROS is 8-hydroxy-2′-deoxyguanosine (8-oxodG) which is a product of oxidative damage of guanine; without repair, adenine can pair incorrectly to 8-oxodG in place of cytosine. 8-oxodG is increased in keratinocytes of patients with SLE exposed to UV [5], in plasma [94], and in circulating immune complexes [35]. On the other hand, the levels of human 8-oxoguanine glycosylase (hOGG1) which is the main enzyme involved in repairing 8-oxodG by BER mechanism are lower in the plasma of patients with SLE (Figure 1) [94]. The above could result in cell death and the binding of anti-DNA antibodies to ROS-denatured DNA [35], because the changes in DNA by ROS increases DNA immunogenicity [95, 96].

Other DNA repairing enzyme polymorphisms or mutations also increase the risk of developing SLE. Three prime repair exonuclease 1 (TREX1) gene mutation, which encodes a potent DNA exonuclease, generates dysfunctional DNA degradation and may result in the accumulation of single- or double-stranded DNA degradation products that could trigger an autoimmune response (76–78). TREX1 has been linked to a spectrum of diseases including SLE, lupus perniosis (Chilblain lupus), and Aicardi-Goutières syndrome (AGS) [97–100]. Furthermore, structural modifications in the DNA structure by oxidative damage reduce efficient degradation by TREX1 [5].

AGS is a pediatric disorder that shares clinical and serologic features and abnormalities described in patients with SLE and interestingly represents a part which has been named monogenic lupus [101–103], that is, a group of monogenic disorders that present with a lupus-like phenotype. AGS is caused by the mutation on any of the 3 domains of H2 ribonuclease (RNase H2) [104]: in TREX1 [105], in the sterile alpha motif domain, and in HD-containing protein 1 (SAMHD1) [106], or adenosine deaminases acting on RNA (ADAR1) [107]. RNase H2 is essential for removing erroneously incorporated ribonucleotides in the genome during DNA replication [108]. Günther et al. showed that SLE patients present mutations in three subunits of this ribonuclease. The authors also reported that misincorporated ribonucleotides persisting in DNA enhanced the formation of UVR-induced CPDs as well as an increase in type I IFN signaling [109].

Further evidencing the potential influence of isolated polymorphisms, a product of the NBS1 gene, nibrin, is a protein involved in double-stranded DNA repair and maintenance of telomeres. The haplotypes Ht1-GGG, Ht2-AAC, and Ht3-AGC of NBS1 have been found to be associated with a lower risk of SLE. However, the haplotypes Ht4-AAG, Ht5-AGG, and Ht8-GGC increase the risk of developing this disease [110].

The presence of autoantibodies against DNA repair proteins has also been reported in SLE patients. Ku is a DNA-binding protein that plays a key role in double-stranded DNA repair. Ku is also involved in the protection of telomeres, in DNA replication, and in regulation of gene transcription. This protein interacts with the DNA ligase IV/XRCC4 complex, WRN (Werner syndrome protein), and poly(ADP-ribose) polymerase 1 (PARP-1). Schild-Poulter et al. found a significantly higher prevalence of anti-Ku antibodies in SLE patients compared to healthy individuals. Additionally, the authors found a higher prevalence of antibodies against WRN and PARP [111].

Also, the nuclear enzyme PARP-1 catalyzes the polyADP-ribosylation of nuclear proteins as an immediate response to DNA damage. It has been associated with repair of UV DNA damage. Also, a decreased synthesis of PARP-1 has been found in lymphocytes isolated from SLE patients [112], as well as in PBMC after exposure to UV compared to healthy controls [113].

According to the above, the deficiency in the damaged DNA response is a factor of great importance for the presence of SLE. Such is the relevance of these deficiencies that only the alteration of a single repair protein can give the characteristic phenotype of SLE. The reviewed articles show the diversity of possible failures in DNA repair that have been associated with the presence of disease. The deficiencies or enzymatic abnormalities found in patients with SLE or in *in vivo* models of the disease may belong to any of the major DNA repair mechanisms: NER, BER, NHEJ, or HR (Figure 1).

## 6. Futuristic Approach Treatments

It is remarkable to us that, in a consistent fashion, defective proteins involved in the DNA repair process are found in SLE patients. As a confirmation if the defect is induced in animal models, it replicates key abnormalities reported in human SLE patients such as nephritis, which is not intuitively connected to genomic integrity. Furthermore, as the DNA reparative process becomes more deficient, it worsens the severity and extension of the inflammatory process. This link between the severity of the genetic abnormality and the disease activity and refractoriness in the animal models clearly opens the possibility that the graveness of immune response abnormality is indeed an epiphenomenon representing the intensity of cellular dysfunction, and also the degree of cellular annoyance escalated as a chronic inflammatory response, which will prime a reactive immune response; this connection has been also explained in our previous paper [36]. The association between the degree of the genetic abnormalities and disease severity in animal models presents a scenario to understand the heterogeneity of the human SLE and to better search for a potential explanation in refractory or grave variants.

Most therapeutic strategies in SLE have been centered in the adaptive immune response and therefore in the interface between T and B cells. In recent years, we have witnessed a significant advance in the therapeutics of inflammatory rheumatic diseases, specially arthritides. Rheumatoid arthritis' therapeutic arsenal now includes several specific biologic targets that, if blocked, improve disease activity and control structural progression in a new dimension; the same can be said to spondyloarthritis, with psoriatic arthritis included. This connection between our improved understanding of the disease pathogenesis and the consequent development of successful therapies has not reached a comparable level of success if we stare at connective tissue diseases including SLE.

Such lack of new target-specific therapies for SLE patients is by no means the consequence of insufficient efforts. A specific PUBMED search shows a total of 48 phase I clinical trials on SLE and 64 on phase II. Although several approaches have been intended, target-specific biological therapies are a pending matter. Abatacept [114, 115] was unsuccessful in controlling renal and extrarenal SLE. Rituximab, originally a hematooncological drug, has proven successful in refractory manifestations of SLE. After this success, B cell-directed agents such as belimumab, epratuzumab, and atacicept [116–118] were considered a tempting possibility. B cell blockade seems a logical step in a disease featuring severe derangement in humoral immunology and in which autoantibodies represent the mediators of tissue damage and perpetuate the inflammatory process. Nevertheless, success beyond that of rituximab has not been achieved with newer agents, and currently only belimumab became a commercial treatment and mostly for patients with mild disease (excluding patients with nephritis, central nervous system involvement, or severe thrombocytopenia), indicating that its direct impact on the pathogenic process is limited and that likely B cell derangement is more of a consequence than an etiologic aspect.

CD4 T-cell depletion on the other hand has proved to be ineffective in rheumatoid arthritis and has not been recently reattempted in SLE, beyond the initial anti-CD4 [119] a long time ago (also unsuccessful), as it was the blockade of T-cell cytokines (such as IFN-*γ*) [120]. This inability to control the disease raises the question whether SLE is actually a disease characterized by autoimmunity (adaptive cell derangement) as the primary event or if autoimmunity is a compensatory consequence of a severe tissular abnormality, and therefore, our therapeutic targets should go beyond attempting immune regulation to focus in the proinflammatory consequences of cell suffering.

IFN-*α* has become an attractive therapeutic target in SLE, since it is considered a crucial pathogenic mediator. IFN-*α* has become an attractive therapeutic target in SLE since it is considered a crucial pathogenic mediator; currently, antibodies against IFN-*α* are being assessed in clinical trials [121]; however, anifrolumab successfully improved patients' disease activity compared to placebo in a phase IIb trial [122] and remains a promising drug. Interestingly, IFN-*α* restingly by plasmacytoid dendritic cells but also by keratinocytes under stress [123, 124] and UV light is one of the stimuli; therefore, we are probably blocking a primary pathogenic mechanism.

Antagonizing DDR as a therapeutic area of opportunity has been considered in cancer, where accumulated DNA damage is central in its pathogenesis [125]. Several drugs target DDR mediators including ATM (ataxia-telangiectasia-mutated) and ATR (ATM and Rad3-related). Those agents are now under evaluation on clinical trials; it would not be surprising if eventually these drugs could have a place on SLE treatment. As a matter of interest, antineoplastic drugs such as cyclophosphamide, azathioprine, and methotrexate are effective in a diversity of disease manifestations in SLE; they all alter nucleic acid dynamics, and mycophenolate mofetil, although not an antineoplastic drug, inhibits the synthesis of guanosine nucleotides [126]. Therefore, it also alters nucleic acids' biology. It is possible that their effect on DDR more than its cytostatic role explains their usefulness. Defining the intimate nature of SLE either as a predominantly autoimmune disease versus a primarily tissular dysfunctional one, and defining the real relevance of the DDR in the pulse of the inflammatory response, could give powerful insights in the strategies to develop novel therapies for patients with SLE.

## 7. Conclusion

The experimental findings presented in this review show that increased DNA damage and deficiencies in enzyme systems to repair it are factors implicated in the pathogenesis of SLE. From our perspective, the pathogenesis of SLE focuses on cellular impairment to repair damaged DNA. This pathogenic process is better understood under the perspective of the danger model and the connection between cellular dysfunctions. In a previous paper, we defined the concept of cellular perennial annoyance as that induced by the chronic impairment of a cell to carry its physiological roles (i.e., explained by genetic defects); this impairment can result in the chronic induction of inflammation and likely a proinflammatory immune response. The specific mechanisms linking cellular dysfunction to the induction of immune responses have been discussed in detail on that article [36].

Physiologically, the keratinocyte irradiated by UV secretes a variety of proteins, some of them related to DNA repair and others with proinflammatory activity. This proinflammatory response allows communication between keratinocyte and local antigen-presenting cells, as well as other adaptive immune response cells. In a scenario where keratinocyte DNA repair mechanisms are defective, the poorly repaired DNA and the compensatory response would result in the accumulation of potentially antigenic nuclear material and autoantibody production. In this way, the continuous effort of the cell to achieve the repair of its genetic material generates a perpetual inflammatory state that could explain the chronic nature of SLE.

Interestingly, several published reports consistently indicate an association between defective DNA repair processes and SLE in humans, as does the presence of SLE-like disease in animal models of defective DNA repair. Furthermore, the more severe the protein deficiency is, the more severe the disease is. These findings also open the possibility that the intensity of the immune response abnormality is indeed an epiphenomenon representing the intensity of cellular dysfunction. Also, the degree of cellular annoyance escalates as a chronic inflammatory response, which will prime a reactive immune response.

## Figures and Tables

**Table 1:** Evidence of increased DNA damage in systemic lupus erythematosus.

Type of sample/origin	Study groups	Methodological strategy	Main findings	Reference
(i) Urine (ii) Venous blood (iii) Peripheral blood mononuclear cells	(i) SLE patients (ii) RA patients (iii) Healthy individuals	(i) Determination of 8-oxodG levels in urine and DNA of immune complexes. (ii) Quantification of DNA base products in monocytes under oxidative stress (H_2_O_2_) and irradiated with UV	(i) 8-oxodG levels were 10^3^ times lower in urine and higher in DNA associated with circulating immune complexes (plasma) in SLE patients (0.38–3.6 pmol 8-oxodG/mg DNA) compared to patients with RA and healthy subjects. (ii) In response to H_2_0_2_, SLE cells showed a rapid conversion of dG to 8-oxodG as well as cells from healthy individuals. However, the rate of removal of the damaged base and viability decreased in SLE cells (*p* < 0.05).	[4, 35]

(i) Peripheral blood mononuclear cells	(i) SLE patients (ii) Patients with SLE and aggregated CVD (cases) (iii) Patients with SLE without aggregated CVD (controls) (iv) Healthy individuals	(i) Comet assay. (ii) Determination of MDA level (iii) Determination of SOD activity	(i) Increased DNA damage in SLE patients cells compared to healthy individuals (ii) Plasma increase of MDA in SLE patients compared to healthy subjects (*p* < 0.0001) (iii) Decrease of SOD activity in SLE patients compared to healthy individuals (*p* = 0.0071) (iv) Less SOD activity in cases with SLE compared to SLE control patients	[69]

(i) Freshly isolated neutrophils (ii) Cultured neutrophils	(i) SLE patients (ii) RA patients (iii) Healthy individuals	(i) Comet assay (ii) Apoptosis evaluation by binding to Annexin V and cell morphology (iii) Repair rates of oxidative DNA damage by formamidopyrimidine DNA glycosylase incorporation in the comet assay	(i) Greater damage in nuclear DNA in neutrophils isolated and cultured from SLE patients (median = 12.5% and 27.3%, resp.) compared to RA patients (median = 9.4%, *p* = 0.002, and 19.3%, *p* = 0.002, resp.) and healthy individuals (median = 8.2%, *p* = 0.003, and 18.7%, *p* = 0.01, resp.). (ii) Higher levels of circulating apoptotic neutrophils in SLE patients compared to RA patients and healthy individuals (iii) Altered ability to repair oxidized DNA in neutrophils in 3 of 5 patients with SLE	[66]

(i) Peripheral blood mononuclear cells	(i) SLE patients (ii) Healthy individuals	(i) Determination of DNA damage and induction of apoptosis in PBMCs exposed to melphalan and cisplatin by quantifying H2AX foci by immunofluorescence and comet assay	(i) Increase in intrinsic DNA damage in SLE patients' cells compared to healthy individuals' cells (Olive Tail Moment units of 15.8 ± 2.3 versus 3.0 ± 1.4 in comet assay *p* < 0.01) (ii) Lower doses of melphalan and cisplatin (9.9 ± 4.8 or 29.8 ± 8.3 *μ*g/ml, resp.) are required to cause apoptosis in SLE cells compared to control cells (32.3 ± 7.7 or 67.7 ± 5.5 *μ*g/ml, resp.) (iii) Greater double-strand DNA breaks in SLE patients' cells than in healthy individuals' cells (*p* < 0.05) (iv) In SLE patients, double-stranded DNA levels correlated with apoptosis levels (*p* < 0.01).	[127]

(i) Peripheral blood mononuclear cells	(i) SLE patients (ii) Healthy individuals	(i) Determination of oxidative lesions in mtDNA by PCR.	(i) Higher levels of mtDNA damage in SLE patients compared to healthy individuals (0.41 lesions/10 kb/string versus 0.10 lesions/10 kb/strand; *p* = 0.002). (ii) SLE patients without major organ affection showed a 20% increase in mtDNA injury levels compared to healthy controls (0.489 lesions versus 0.101; *p* = 0.003). (iii) SLE patients with major organ affection showed a tendency to have lower levels of mtDNA. (iv) The disease degree damage correlated negatively with mtDNA levels (*p* = 0.034). (v) The number of mtDNA lesions correlated positively with the duration of the disease (*p* = 0.034).	[70]

DNA: deoxyribonucleic acid; mtDNA: mitochondrial DNA; RA: rheumatoid arthritis; PBMC: peripheral blood mononuclear cells; CVD: cardiovascular disease; SLE: systemic lupus erythematosus; MDA: malondialdehyde; PCR: polymerase chain reaction; UV: ultraviolet radiation; SOD: superoxide dismutase; 8-oxodG: 8-hydroxy-2′-deoxyguanosine.

**Table 2:** Evidence of deficiency DNA damage repair in systemic lupus erythematosus.

Repair molecule(s) analyzed	*Type of sample/origin*	Study groups	Methodological strategy	Main findings	Reference
PARP	(i) *Peripheral blood lymphocytes*	(i) SLE patients (ii) RA patients (iii) Healthy individuals	(i) Measurement of synthesis and degradation of PARP by incorporation of 3H-labeled NAD from acid precipitated cell counts.	(i) Greater decrease (70%) in PARP synthesis in lupus lymphocytes compared to RA and healthy individuals (*p* < 0.01).	[112]

XRCC1	(i) *DNA extracted from peripheral blood lymphocytes*	(i) SLE patients (ii) Healthy individuals	(i) Polymorphism analysis of a single-nucleotide rs1799782 (Arg > Trp codon 194) and rs25487 (Arg > Gln codon 399) of the XRCC1 gene by PCR-RFLP.	(i) Increased frequency of Arg > Gln polymorphism 399 in SLE patients compared to healthy individuals (*p* < 0.01; OR: 1.80; 95% CI: 1.17–2.75). (ii) The presence of this polymorphism was associated more frequently to the presentation of photosensitivity and malar rash (*p* < 0.001; OR: 3.4; 95% CI: 1.8–6.4).	[90]

PARP	(i) *PBMC*	(i) SLE patients (ii) LCSSc patients (iii) dcSSc patients (iv) Healthy individuals	(i) Analysis of PARP activity in PBMC irradiated with UV-C (280 nm) by measuring NAD concentrations by HPLC	(i) SLE cells UV-irradiated showed a decreased PARP activity compared to irradiated cells from control individuals (*p* < 0.05). (ii) Activation of PARP decreased according to an increasing SLEDAI score: 77% for patients with 2–9 score, 71% for a 10–14 score, 22% for a 17–23 score, and 15% for a 24–28 score.	[113]

NBS1	(i) *DNA extracted from peripheral blood leukocytes*	(i) Taiwanese SLE patients (ii) Healthy Taiwanese	(i) Analysis of the distribution of genotypes and allele frequencies for the polymorphisms of the NBS1 gene detected by TaqMan (R) genotyping.	(i) Individuals with Ht1-GGG haplotypes (SLE: 21.75% versus controls: 51.98%, *p* < 0.0019), Ht2-AAC (SLE: 11.79% versus controls: 30.67%, *p* < 0.001), and Ht3-AGC (SLE: 6.63% versus controls: 17.05%, *p* < 0.001) of the NBS1 gene present a lower risk of presenting SLE. (ii) Individuals with Ht4-AAG haplotypes (SLE: 23.04% versus controls: 0.29%, *p* < 0.001), Ht5-AGG (SLE: 12.58% versus controls: 0.01%, *p* < 0.001), and Ht8-GGC SLE: 20.93% versus controls: 0.00%, *p* < 0.001) showed an increased risk of presenting this disease.	[110]

DNase 1	(i) *Serums* (ii) *Renal biopsies*	(i) DNase 1 +/− (ii) DNase 1 −/− mice (iii) Wild type mice (WT)	(i) Generation of a DNase 1 deficiency murine model by exon deletion of the DNase allele (ii) Immunofluorescence evaluation of ANA levels (iii) Histopathological analysis of tissues using H&E or PAS	(i) DNase 1-deficient mice showed classic SLE symptoms, including elevated levels of ANAs (WT: 35% versus DNase 1 −/−: 73%, *p* = 0.013) and glomerulonephritis (WT: 0% versus DNase 1 −/− 19% *p* = 0.037).	[89]

DNase 1	(i) *Serum*	(i) SLE patients (i) SLE patients and glomerulonephritis (i) Healthy individuals	(i) Measurement of DNase 1 activity by the SRED method.	(i) Decreased ADNAsa1 activity in the serum of patients with SLE and with aggregated glomerulonephritis (7 ± 0 ng/ml) compared to controls (16 ± 5.5 ng/ml) and females (14.2 ± 6.5 ng/ml).	[89]

DNA repair genes	(i) *RNA extracted from peripheral blood cells (neutrophils and lymphocytes)*	(i) SLE patients (ii) Healthy individuals	(i) DNA microarray analysis	(i) 4213 genes were differentially expressed in peripheral blood cells from SLE patients compared to healthy individuals. (ii) 2329 genes were upregulated, which were mainly associated with the immune response. (iii) 1884 genes involved in DNA repair and in ATP synthesis were expressed negatively.	[86]

53BP1, SMC1, S phase control point, Fanconi D2 protein, ATM, and nonhomologous DNA-binding proteins.	(i) *B lymphoblastoid cell lines obtained from blood samples*	(i) SLE pediatric patients (ii) Control patients with ataxia telangiectasia (iii) WT mouse control cells	(i) Determination of repair and recognition activity for double-stranded DNA breaks through 9 trials: (1) NCA, (2) CSA, (3, 4) irradiation-induced foci formation by measuring the *γ*-H2AX and 53BP1 proteins, (5) kinetics of SMC1 phosphorylation, (6) incorporation of postradiation bromodeoxyuridine to assess the integrity of the S-phase control point, (7) monoubiquitination of the Fanconi D2 protein, (8) expression of the ATM protein, and (9) expression and function of nonhomologous DNA-binding proteins.	(i) 3 of the 9 trials revealed abnormal patterns in response to radiation-induced DNA damage. (ii) 2 of 16 lymphoblastoid cell lines showed an extension in SMC1 phosphorylation.	[87]

POLB	(i) *Serum* (ii) *Skin biopsies* (iii) *Kidney and spleen cuts*	(i) Pol *β* mice deficient in Pol *β* activity (ii) WT control mice	(i) Construction of a POLB mouse model using directed gene disruption, which induced the encoding of an enzyme with slow DNA polymerase activity (ii) Immunofluorescence evaluation of ANA levels (iii) Histopathological analysis of tissues using H&E or PAS	(i) The mouse that expressed the hypomorphic POLB allele developed pathological features very similar to those present in SLE compared to WT mice, including increased levels of immune complexes in glomeruli, elevated levels of serum ANAs, dermatitis, glomerulonephritis, and cervical lymphadenopathy with infiltrate of T and B lymphocytes.	[88]

hOGG1	(i) *Plasma* (ii) *Peripheral blood leukocytes*	(i) SLE patients (ii) Healthy individuals	(i) ELISA determination of plasma levels of 8-oxodG (ii) Calculation of the number of mtDNA copies by PCR to detect transcription levels of specific genes (8-oxodG repair enzymes, antioxidant enzymes, proteins related to mitochondrial biogenesis, and glycolytic enzymes).	(i) Increased plasma levels of 8-oxodG in SLE patients (*p* < 0.01) (ii) Lower expression of genes encoding hOGG1 (*p* < 0.01), antioxidant enzymes (*p* < 0.05), proteins related to mitochondrial biogenesis (*p* < 0.05), and glycolytic enzymes (*p* < 0.05) in lupus leukocytes compared to healthy individuals (iii) In SLE patients, the increase in plasma levels of 8-oxodG correlated positively with an increase in leukocyte gene expression of genes encoding hOGG1 (*p* < 0.05), antioxidant enzymes (*p* < 0.05), proteins related to mitochondrial biogenesis (*p* < 0.05), and glycolytic enzymes (*p* < 0.05).	[2, 94]

XRCC5 XRCC6 XRCC7	(i) *DNA extracted from blood samples*	(i) SLE patients (ii) Healthy individuals	(i) Genotyping of XRCC5 for the VNTR, and XRCC6-61C> G and XRCC7 6721G> T polymorphisms by PCR and PCR-RFLP, respectively	(i) The presence of the XRCC7 G allele increased the frequency of SLE (*p* = 0.04). (ii) The frequency of the 1R (*p* = 0.003), 2R (*p* < 0.001), and 3R (*p* = 0.041) alleles of the VNTR XRCC5 polymorphism was found to be significantly decreased in SLE patients compared to the control group. (iii) The frequency of the 0R allele (*p* = 0.032) and 2R allele (*p* = 0.024) increased in patients with malar rash. (iv) A decreased presence of the 2R allele was found in patients with a positive ANA test (*p* = 0.03).	[91]

N-ras, *γ*H2AX, Rad51, 84 for DNA damage signaling genes	(i) *CMSP*	(i) SLE patients (ii) Healthy individuals	(i) Induction of DNA damage and apoptosis with different doses of melphalan (ii) Nucleotide cleavage evaluation by Western blot at different times (monofunctional binding of melphalan to a single DNA site (monoadducts) (iii) Immunofluorescence and confocal laser scanning microscopy evaluation of double-strand DNA rupture repair (iv) PCR detection of the genes involved in the DNA damage response	(i) Defects in DNA repair were found by nucleotide cleavage and DNA repair by double-stranded rupture in SLE patients. (ii) Higher levels of DNA damage were found in patients with lupus nephritis than in those with quiescent SLE (*p* < 0.038) and healthy individuals (*p* < 0.001). (iii) The rate of apoptosis induced by melphalan was higher in SLE than in control subjects (*p* < 0.001) and inversely correlated with deficiency in DNA repair. (iv) The genes involved in DNA signaling and repair pathways were significantly less expressed in SLE than in control individuals. However, the genes involved in apoptosis were more expressed.	[71]

ARNase H2	(i) *Fibroblasts isolated from skin* (ii) *Blood*	(i) SLE patients (ii) AGS patients (iii) Healthy individuals	(i) Mutation analysis of the 3 subunits of RNase H2 (RNASEH2A, RNA-SEH2B, and RNASEH2C) by PCR in blood samples (ii) Exposure of fibroblasts to UV (250 nm) and analysis of genomic DNA by Southwestern blot (iii) Detection of double-strand DNA breaks and the presence of CPDs in skin biopsies by immunohistochemistry and immunofluorescence	(i) An altered function of RNase H2 correlated with the risk of presenting SLE. (ii) A mild failure conferred a relative risk of 1.6 times (OR, 1.69, *p* = 0.03), while a severe condition in RNase H2 increased irrigation to 3.8 times (OR, 3.94, *p* = 0.003). (iii) Cutaneous lupus and photosensitivity were the predominant symptoms in SLE patients who had mutations in RNASEH2. (iv) An increase in CPDs in RNase H2-deficient fibroblasts was found in SLE (*p* < 0.05) and AGS (*p* < 0.001) patients compared to control fibroblasts. (v) Injured skin of patients with mutations in RNASEH2B and RNASEH2C had a higher expression of IFN-LES-induced proteins *p* < 0.05, AGS *p* < 0.01, and controls *p* < 0.001	[109]

ANA: antinuclear antibody; DNA: deoxyribonucleic acid; mtDNA: mitochondrial DNA; RA: rheumatoid arthritis; AGS: Aicardi-Goutières syndrome; PBMC: peripheral blood mononuclear cells; CPDs: cyclobutane pyrimidine dimers; CSA: colony survival assay; ELISA: enzyme-linked immunosorbent assay; ESD: diffuse systemic sclerosis; LSSC: limited systemic sclerosis; Gy: Gray units; H&E: hematoxylin and eosin; hOGG1: 8-oxoguanine DNA glycosylase; HPLC: high-performance liquid chromatography; NBS: Nijmegen's syndrome; NCA: neutral comet assay; PARP: poly-ADP ribose polymerase; PAS: periodic acid and Schiff; PCR: polymerase chain reaction; PCR-RFLP: polymerase chain reaction-restriction fragment length polymorphisms; Pol *β*: polymerase beta; UV: ultraviolet radiation; SLEDAI: systemic lupus erythematosus disease activity index; SMC1: structural maintenance protein 1 of chromosomes; SRED: single radial enzyme-diffusion; VNTR: variable number of tandem repeats; XRCC: X-ray repair cross-complementing protein; 8-oxodG: 8-hydroxy-2′-deoxyguanosine.

## References

[1] Hejazi E. Z., Werth V. P. (2016). Cutaneous lupus erythematosus: an update on pathogenesis, diagnosis and treatment. *American Journal of Clinical Dermatology*.

[2] Hersh A. O., Arkin L. M., Prahalad S. (2016). Immunogenetics of cutaneous lupus erythematosus. *Current Opinion in Pediatrics*.

[3] Petri M., Orbai A.-M., Alarcón G. S. (2012). Derivation and validation of the systemic lupus international collaborating clinics classification criteria for systemic lupus erythematosus. *Arthritis and Rheumatism*.

[4] Scholtissek B., Zahn S., Maier J. (2017). Immunostimulatory endogenous nucleic acids drive the lesional inflammation in cutaneous lupus erythematosus. *The Journal of Investigative Dermatology*.

[5] Gehrke N., Mertens C., Zillinger T. (2013). Oxidative damage of DNA confers resistance to cytosolic nuclease TREX1 degradation and potentiates STING-dependent immune sensing. *Immunity*.

[6] Li T., Chen Z. J. (2018). The cGAS–cGAMP–STING pathway connects DNA damage to inflammation, senescence, and cancer. *The Journal of Experimental Medicine*.

[7] Kawanishi S., Ohnishi S., Ma N., Hiraku Y., Murata M. (2017). Crosstalk between DNA damage and inflammation in the multiple steps of carcinogenesis. *International Journal of Molecular Sciences*.

[8] Eliopoulos A. G., Havaki S., Gorgoulis V. G. (2016). DNA damage response and autophagy: a meaningful partnership. *Frontiers in Genetics*.

[9] Nakad R., Schumacher B. (2016). DNA damage response and immune defense: links and mechanisms. *Frontiers in Genetics*.

[10] Rancoule C., Vallard A., Guy J.-B. (2017). Impairment of DNA damage response and cancer. *Bulletin du Cancer*.

[11] Gorgoulis V. G., Pefani D. E., Pateras I. S., Trougakos I. P. (2018). Integrating the DNA damage and protein stress responses during cancer development and treatment. *The Journal of Pathology*.

[12] Carrassa L., Damia G. (2017). DNA damage response inhibitors: mechanisms and potential applications in cancer therapy. *Cancer Treatment Reviews*.

[13] Morales A. J., Carrero J. A., Hung P. J. (2017). A type I IFN-dependent DNA damage response regulates the genetic program and inflammasome activation in macrophages. *eLife*.

[14] Moretti J., Blander J. M. (2017). Cell-autonomous stress responses in innate immunity. *Journal of Leukocyte Biology*.

[15] Soria-Valles C., López-Soto A., Osorio F. G., López-Otín C. (2017). Immune and inflammatory responses to DNA damage in cancer and aging. *Mechanisms of Ageing and Development*.

[16] Budden T., Bowden N. A. (2013). The role of altered nucleotide excision repair and UVB-induced DNA damage in melanomagenesis. *International Journal of Molecular Sciences*.

[17] Matsumura Y., Ananthaswamy H. N. (2004). Toxic effects of ultraviolet radiation on the skin. *Toxicology and Applied Pharmacology*.

[18] Afaq F., Mukhtar H. (2001). Effects of solar radiation on cutaneous detoxification pathways. *Journal of Photochemistry and Photobiology B: Biology*.

[19] Mitchell D. (2006). Revisiting the photochemistry of solar UVA in human skin. *Proceedings of the National Academy of Sciences of the United States of America*.

[20] Svobodova A., Walterova D., Vostalova J. (2006). Ultraviolet light induced alteration to the skin. *Biomedical Papers*.

[21] Pattison D. I., Davies M. J. (2006). Actions of ultraviolet light on cellular structures. *Cancer: Cell Structures, Carcinogens and Genomic Instability*.

[22] Rastogi R. P., Richa, Kumar A., Tyagi M. B., Sinha R. P. (2010). Molecular mechanisms of ultraviolet radiation-induced DNA damage and repair. *Journal of Nucleic Acids*.

[23] Mouret S., Baudouin C., Charveron M., Favier A., Cadet J., Douki T. (2006). Cyclobutane pyrimidine dimers are predominant DNA lesions in whole human skin exposed to UVA radiation. *Proceedings of the National Academy of Sciences of the United States of America*.

[24] Douki T., Reynaud-Angelin A., Cadet J., Sage E. (2003). Bipyrimidine photoproducts rather than oxidative lesions are the main type of DNA damage involved in the genotoxic effect of solar UVA radiation. *Biochemistry*.

[25] Schuch A. P., Moreno N. C., Schuch N. J., Menck C. F. M., Garcia C. C. M. (2017). Sunlight damage to cellular DNA: focus on oxidatively generated lesions. *Free Radical Biology and Medicine*.

[26] Ikehata H., Ono T. (2011). The mechanisms of UV mutagenesis. *Journal of Radiation Research*.

[27] Pfeifer G. P. (1997). Formation and processing of UV photoproducts: effects of DNA sequence and chromatin environment. *Photochemistry and Photobiology*.

[28] You Y. H., Lee D. H., Yoon J. H., Nakajima S., Yasui A., Pfeifer G. P. (2001). Cyclobutane pyrimidine dimers are responsible for the vast majority of mutations induced by UVB irradiation in mammalian cells. *The Journal of Biological Chemistry*.

[29] Jiang Y., Rabbi M., Kim M. (2009). UVA generates pyrimidine dimers in DNA directly. *Biophysical Journal*.

[30] Cadet J., Courdavault S., Ravanat J.-L., Douki T. (2005). UVB and UVA radiation-mediated damage to isolated and cellular DNA. *Pure and Applied Chemistry*.

[31] Cadet J., Sage E., Douki T. (2005). Ultraviolet radiation-mediated damage to cellular DNA. *Mutation Research*.

[32] Rünger T. M., Farahvash B., Hatvani Z., Rees A. (2012). Comparison of DNA damage responses following equimutagenic doses of UVA and UVB: a less effective cell cycle arrest with UVA may render UVA-induced pyrimidine dimers more mutagenic than UVB-induced ones. *Photochemical & Photobiological Sciences*.

[33] Salimi S., Mohammadoo-khorasani M., Tabatabai E., Sandoughi M., Zakeri Z., Naghavi A. (2014). *XRCC1* Arg399Gln and Arg194Trp polymorphisms and risk of systemic lupus erythematosus in an Iranian population: a pilot study. *BioMed Research International*.

[34] Evans M. D., Cooke M. S., Akil M., Samanta A., Lunec J. (2000). Aberrant processing of oxidative DNA damage in systemic lupus erythematosus. *Biochemical and Biophysical Research Communications*.

[35] Lunec J., Herbert K., Blount S., Griffiths H. R., Emery P. (1994). 8-Hydroxydeoxyguanosine. A marker of oxidative DNA damage in systemic lupus erythematosus. *FEBS Letters*.

[36] Pacheco-Tena C., González-Chávez S. A. (2015). The danger model approach to the pathogenesis of the rheumatic diseases. *Journal of Immunology Research*.

[37] Tebbe B., Orfanos C. E. (1997). Epidemiology and socioeconomic impact of skin disease in lupus erythematosus. *Lupus*.

[38] Barbhaiya M., Costenbader K. H. (2016). Environmental exposures and the development of systemic lupus erythematosus. *Current Opinion in Rheumatology*.

[39] Lan C.-C. E., Wu C. S., Huang S. M. (2016). Irradiance-dependent UVB photocarcinogenesis. *Scientific Reports*.

[40] Dong Y., Zhang Y., Xia L. (2017). The deposition of anti-DNA IgG contributes to the development of cutaneous lupus erythematosus. *Immunology Letters*.

[41] Reich A., Marcinow K., Bialynicki-Birula R. (2011). The lupus band test in systemic lupus erythematosus patients. *Therapeutics and Clinical Risk Management*.

[42] Elbendary A., Zhou C., Valdebran M. (2016). Specificity of granular IgM deposition in folliculosebaceous units and sweat gland apparatus in direct immunofluorescence (DIF) of lupus erythematosus. *Journal of the American Academy of Dermatology*.

[43] Furukawa F. (1999). Antinuclear antibody-keratinocyte interactions in photosensitive cutaneous lupus erythematosus. *Histology and Histopathology*.

[44] LeFeber W. P., Norris D. A., Ryan S. R. (1984). Ultraviolet light induces binding of antibodies to selected nuclear antigens on cultured human keratinocytes. *Journal of Clinical Investigation*.

[45] Oke V., Vassilaki I., Espinosa A. (2009). High Ro52 expression in spontaneous and UV-induced cutaneous inflammation. *The Journal of Investigative Dermatology*.

[46] Herrera-Esparza R., Villalobos R., Bollain-y-Goytia J. J. (2006). Apoptosis and redistribution of the Ro autoantigen in Balb/c mouse like in subacute cutaneous lupus erythematosus. *Clinical and Developmental Immunology*.

[47] Jones S. K. (1992). Ultraviolet radiation (UVR) induces cell-surface Ro/SSA antigen expression by human keratinocytes in vitro: a possible mechanism for the UVR induction of cutaneous lupus lesions. *The British Journal of Dermatology*.

[48] Lawley W., Doherty A., Denniss S. (2000). Rapid lupus autoantigen relocalization and reactive oxygen species accumulation following ultraviolet irradiation of human keratinocytes. *Rheumatology*.

[49] Sánchez-Rodríguez S. H., Herrera-van Oostdam D., Avalos-Díaz E., Herrera-Esparza R. (2007). Ro60 and La ribonucleoproteins become self-aggregated by cell stress. *Reumatismo*.

[50] Unterholzner L., Keating S. E., Baran M. (2010). IFI16 is an innate immune sensor for intracellular DNA. *Nature Immunology*.

[51] Mondini M., Vidali M., Airo P. (2007). Role of the interferon-inducible gene IFI16 in the etiopathogenesis of systemic autoimmune disorders. *Annals of the New York Academy of Sciences*.

[52] Gugliesi F., de Andrea M., Mondini M. (2010). The proapoptotic activity of the interferon-inducible gene IFI16 provides new insights into its etiopathogenetic role in autoimmunity. *Journal of Autoimmunity*.

[53] Choubey D., Panchanathan R. (2016). IFI16, an amplifier of DNA-damage response: role in cellular senescence and aging-associated inflammatory diseases. *Ageing Research Reviews*.

[54] Costa S., Borgogna C., Mondini M. (2011). Redistribution of the nuclear protein IFI16 into the cytoplasm of ultraviolet B-exposed keratinocytes as a mechanism of autoantigen processing. *British Journal of Dermatology*.

[55] Caneparo V., Cena T., de Andrea M. (2013). Anti-IFI16 antibodies and their relation to disease characteristics in systemic lupus erythematosus. *Lupus*.

[56] Kuechle M. K., Elkon K. B. (2007). Shining light on lupus and UV. *Arthritis Research & Therapy*.

[57] Bijl M., Kallenberg C. G. M. (2006). Ultraviolet light and cutaneous lupus. *Lupus*.

[58] Caricchio R., McPhie L., Cohen P. L. (2003). Ultraviolet B radiation-induced cell death: critical role of ultraviolet dose in inflammation and lupus autoantigen redistribution. *The Journal of Immunology*.

[59] Ansel J. C., Mountz J., Steinberg A. D., DeFabo E., Green I. (1985). Effects of UV radiation on autoimmune strains of mice: increased mortality and accelerated autoimmunity in BXSB male mice. *Journal of Investigative Dermatology*.

[60] Zamansky G. B. (1985). Sunlight-induced pathogenesis in systemic lupus erythematosus. *The Journal of Investigative Dermatology*.

[61] Pisetsky D. S. (2016). Anti-DNA antibodies — quintessential biomarkers of SLE. *Nature Reviews Rheumatology*.

[62] Blount S., Lunec J., Griffiths H., Herbert K., Isenberg D. (1994). Binding of anti-DNA antibodies to oxidatively damaged DNA in spouses and relatives of patients with systemic lupus erythematosus. *Immunology Letters*.

[63] Ara J., Ali R. (1992). Reactive oxygen species modified DNA fragments of varying size are the preferred antigen for human anti-DNA autoantibodies. *Immunology Letters*.

[64] Ahsan H., Ali A., Ali R. (2003). Oxygen free radicals and systemic autoimmunity. *Clinical and Experimental Immunology*.

[65] Alam K., Ali R. (1999). Human anti-DNA autoantibodies and induced antibodies against ROS-modified-DNA show similar antigenic binding characteristics. *Biochemistry and Molecular Biology International*.

[66] McConnell J. R., Crockard A. D., Cairns A. P., Bell A. L. (2002). Neutrophils from systemic lupus erythematosus patients demonstrate increased nuclear DNA damage. *Clinical and Experimental Rheumatology*.

[67] Benke P. J., Belmar P. (1991). Phytohemagglutinin-stimulated lymphocytes from patients with systemic lupus erythematosus demonstrate DNA damage. *Metabolism*.

[68] Carrillo-Alascio P. L., Sabio J. M., Núñez-Torres M. I. (2009). In-vitro radiosensitivity in patients with systemic lupus erythematosus. *Lupus*.

[69] Zineldeen D. H., Keshk W. A., Ghazy A. H., El-Barbary A. M. (2016). Sucrose non-fermenting AMPK related kinase/Pentraxin 3 and DNA damage axis: a gateway to cardiovascular disease in systemic lupus erythematosus among Egyptian patients. *Annals of Clinical Biochemistry*.

[70] López-López L., Nieves-Plaza M., del R Castro M. (2014). Mitochondrial DNA damage is associated with damage accrual and disease duration in patients with systemic lupus erythematosus. *Lupus*.

[71] Souliotis V. L., Vougas K., Gorgoulis V. G., Sfikakis P. P. (2016). Defective DNA repair and chromatin organization in patients with quiescent systemic lupus erythematosus. *Arthritis Research & Therapy*.

[72] Richa, Sinha R. P., Häder D.-P. (2015). Physiological aspects of UV-excitation of DNA. *Photoinduced Phenomena in Nucleic Acids II*.

[73] Krokan H. E., Bjoras M. (2013). Base excision repair. *Cold Spring Harbor Perspectives in Biology*.

[74] Yasui A. (2013). Alternative excision repair pathways. *Cold Spring Harbor Perspectives in Biology*.

[75] Thoma F. (1999). Light and dark in chromatin repair: repair of UV-induced DNA lesions by photolyase and nucleotide excision repair. *The EMBO Journal*.

[76] Leibeling D., Laspe P., Emmert S. (2006). Nucleotide excision repair and cancer. *Journal of Molecular Histology*.

[77] Hanawalt P. C. (2002). Subpathways of nucleotide excision repair and their regulation. *Oncogene*.

[78] Fousteri M., Vermeulen W., van Zeeland A. A., Mullenders L. H. F. (2006). Cockayne syndrome A and B proteins differentially regulate recruitment of chromatin remodeling and repair factors to stalled RNA polymerase II in vivo. *Molecular Cell*.

[79] Kulaksiz G., Reardon J. T., Sancar A. (2005). Xeroderma pigmentosum complementation group E protein (XPE/DDB2): purification of various complexes of XPE and analyses of their damaged DNA binding and putative DNA repair properties. *Molecular and Cellular Biology*.

[80] Keeney S., Chang G. J., Linn S. (1993). Characterization of a human DNA damage binding protein implicated in xeroderma pigmentosum E. *The Journal of Biological Chemistry*.

[81] Pawlowska E., Wysokinski D., Blasiak J. (2016). Nucleotide excision repair and vitamin D—relevance for skin cancer therapy. *International Journal of Molecular Sciences*.

[82] Emmert S., Slor H., Busch D. B. (2002). Relationship of neurologic degeneration to genotype in three xeroderma pigmentosum group G patients. *The Journal of Investigative Dermatology*.

[83] Petit C., Sancar A. (1999). Nucleotide excision repair: from E. coli to man. *Biochimie*.

[84] de Boer J., Hoeijmakers J. H. J. (2000). Nucleotide excision repair and human syndromes. *Carcinogenesis*.

[85] Farrell A. W., Halliday G. M., Lyons J. G. (2011). Chromatin structure following UV-induced DNA damage—repair or death?. *International Journal of Molecular Sciences*.

[86] Lee H.-M., Sugino H., Aoki C., Nishimoto N. (2011). Underexpression of mitochondrial-DNA encoded ATP synthesis-related genes and DNA repair genes in systemic lupus erythematosus. *Arthritis Research & Therapy*.

[87] Davies R. C., Pettijohn K., Fike F. (2012). Defective DNA double-strand break repair in pediatric systemic lupus erythematosus. *Arthritis & Rheumatism*.

[88] Senejani A. G., Liu Y., Kidane D. (2014). Mutation of POLB causes lupus in mice. *Cell Reports*.

[89] Möröy T., Napirei M., Karsunky H., Zevnik B., Stephan H., Mannherz H. G. (2000). Features of systemic lupus erythematosus in Dnase1-deficient mice. *Nature Genetics*.

[90] Lin Y.-J., Wan L., Huang C. M. (2009). Polymorphisms in the DNA repair gene XRCC1 and associations with systemic lupus erythematosus risk in the Taiwanese Han Chinese population. *Lupus*.

[91] Jahantigh D., Salimi S., Mousavi M. (2015). Association between functional polymorphisms of DNA double-strand breaks in repair genes XRCC5, XRCC6 and XRCC7 with the risk of systemic lupus erythematosus in South East Iran. *DNA and Cell Biology*.

[92] Warchoł T., Mostowska A., Lianeri M., Łącki J. K., Jagodziński P. P. (2012). XRCC1 Arg399Gln gene polymorphism and the risk of systemic lupus erythematosus in the Polish population. *DNA and Cell Biology*.

[93] Zhang M.-Y., Yang X.-K., Lv T.-T. (2018). Meta-analysis of associations between XRCC1 gene polymorphisms and susceptibility to systemic lupus erythematosus and rheumatoid arthritis. *International Journal of Rheumatic Diseases*.

[94] Lee H.-T., Lin C. S., Lee C. S., Tsai C. Y., Wei Y. H. (2014). Increased 8-hydroxy-2′-deoxyguanosine in plasma and decreased mRNA expression of human 8-oxoguanine DNA glycosylase 1, anti-oxidant enzymes, mitochondrial biogenesis-related proteins and glycolytic enzymes in leucocytes in patients with systemic lupus erythematosus. *Clinical and Experimental Immunology*.

[95] Waris G., Alam K. (2004). Immunogenicity of superoxide radical modified-DNA: studies on induced antibodies and SLE anti-DNA autoantibodies. *Life Sciences*.

[96] Cooke M. S., Mistry N., Wood C., Herbert K. E., Lunec J. (1997). Immunogenicity of DNA damaged by reactive oxygen species—implications for anti-DNA antibodies in lupus. *Free Radical Biology & Medicine*.

[97] Grieves J. L., Fye J. M., Harvey S., Grayson J. M., Hollis T., Perrino F. W. (2015). Exonuclease TREX1 degrades double-stranded DNA to prevent spontaneous lupus-like inflammatory disease. *Proceedings of the National Academy of Sciences of the United States of America*.

[98] Rivas-Larrauri F., Yamazaki-Nakashimada M. A. (2016). Systemic lupus erythematosus: is it one disease?. *Reumatología Clínica*.

[99] Sugiura K., Takeichi T., Kono M. (2012). Severe chilblain lupus is associated with heterozygous missense mutations of catalytic amino acids or their adjacent mutations in the exonuclease domains of 3′-repair exonuclease 1. *The Journal of Investigative Dermatology*.

[100] Lee-Kirsch M. A., Gong M., Chowdhury D. (2007). Mutations in the gene encoding the 3′-5’ DNA exonuclease TREX1 are associated with systemic lupus erythematosus. *Nature Genetics*.

[101] Lo M. S. (2016). Monogenic lupus. *Current Rheumatology Reports*.

[102] Kim H., Sanchez G. A. M., Goldbach-Mansky R. (2016). Insights from Mendelian interferonopathies: comparison of CANDLE, SAVI with AGS, monogenic lupus. *Journal of Molecular Medicine*.

[103] Batu E. D. (2018). Monogenic systemic lupus erythematosus: insights in pathophysiology. *Rheumatology International*.

[104] Crow Y. J., Leitch A., Hayward B. E. (2006). Mutations in genes encoding ribonuclease H2 subunits cause Aicardi-Goutières syndrome and mimic congenital viral brain infection. *Nature Genetics*.

[105] Crow Y. J., Hayward B. E., Parmar R. (2006). Mutations in the gene encoding the 3′-5’ DNA exonuclease TREX1 cause Aicardi-Goutières syndrome at the AGS1 locus. *Nature Genetics*.

[106] Rice G. I., Bond J., Asipu A. (2009). Mutations involved in Aicardi-Goutières syndrome implicate SAMHD1 as regulator of the innate immune response. *Nature Genetics*.

[107] Rice G. I., Kasher P. R., Forte G. M. A. (2012). Mutations in ADAR1 cause Aicardi-Goutières syndrome associated with a type I interferon signature. *Nature Genetics*.

[108] Pendergraft W. F., Means T. K. (2015). AGS, SLE, and *RNASEH2* mutations: translating insights into therapeutic advances. *Journal of Clinical Investigation*.

[109] Günther C., Kind B., Reijns M. A. M. (2015). Defective removal of ribonucleotides from DNA promotes systemic autoimmunity. *The Journal of Clinical Investigation*.

[110] Lin Y.-J., Lan Y. C., Wan L. (2010). The *NBS1* genetic polymorphisms and the risk of the systemic lupus erythematosus in Taiwanese patients. *Journal of Clinical Immunology*.

[111] Schild-Poulter C., Su A., Shih A. (2008). Association of autoantibodies with Ku and DNA repair proteins in connective tissue diseases. *Rheumatology*.

[112] Sibley J. T., Haug B. L., Lee J. S. (1989). Altered metabolism of poly(ADP-ribose) in the peripheral blood lymphocytes of patients with systemic lupus erythematosus. *Arthritis and Rheumatism*.

[113] Cerboni B., Morozzi G., Galeazzi M. (2009). Poly(ADP-ribose) polymerase activity in systemic lupus erythematosus and systemic sclerosis. *Human Immunology*.

[114] The ACCESS Trial Group (2014). Treatment of lupus nephritis with abatacept: the Abatacept and Cyclophosphamide Combination Efficacy and Safety Study. *Arthritis & Rheumatology*.

[115] Furie R., Nicholls K., Cheng T. T. (2014). Efficacy and safety of abatacept in lupus nephritis: a twelve-month, randomized, double-blind study. *Arthritis & Rheumatology*.

[116] Pena-Rossi C., Nasonov E., Stanislav M. (2009). An exploratory dose-escalating study investigating the safety, tolerability, pharmacokinetics and pharmacodynamics of intravenous atacicept in patients with systemic lupus erythematosus. *Lupus*.

[117] Stohl W., Merrill J. T., Looney R. J. (2015). Treatment of systemic lupus erythematosus patients with the BAFF antagonist “peptibody” blisibimod (AMG 623/A-623): results from randomized, double-blind phase 1a and phase 1b trials. *Arthritis Research & Therapy*.

[118] Hoffman R. W., Merrill J. T., Alarcón-Riquelme M. M. E. (2017). Gene expression and pharmacodynamic changes in 1,760 systemic lupus erythematosus patients from two phase III trials of BAFF blockade with tabalumab. *Arthritis & Rheumatology*.

[119] Hiepe F., Volk H. D., Apostoloff E., Baehr R. V., Emmrich F. (1991). Treatment of severe systemic lupus erythematosus with anti-CD4 monoclonal antibody. *The Lancet*.

[120] Boedigheimer M. J., Martin D. A., Amoura Z. (2017). Safety, pharmacokinetics and pharmacodynamics of AMG 811, an anti-interferon-*γ* monoclonal antibody, in SLE subjects without or with lupus nephritis. *Lupus Science & Medicine*.

[121] Kalunian K. C., Merrill J. T., Maciuca R. (2015). A phase II study of the efficacy and safety of rontalizumab (rhuMAb interferon-*α*) in patients with systemic lupus erythematosus (ROSE). *Annals of the Rheumatic Diseases*.

[122] Furie R., Khamashta M., Merrill J. T. (2017). Anifrolumab, an anti–interferon-*α* receptor monoclonal antibody, in moderate-to-severe systemic lupus erythematosus. *Arthritis & Rheumatology*.

[123] Robinson E. S., Werth V. P. (2015). The role of cytokines in the pathogenesis of cutaneous lupus erythematosus. *Cytokine*.

[124] Livden J. K., Nilsen R., Bjerke J. R., Matre R. (1989). In situ localization of interferons in psoriatic lesions. *Archives of Dermatological Research*.

[125] Bernstein C., Prasad A. R., Nfonsam V., Bernstein H. DNA damage, DNA repair and cancer. *DNA Damage DNA Repair Cancer*.

[126] Allison A. C., Eugui E. M. (2000). Mycophenolate mofetil and its mechanisms of action. *Immunopharmacology*.

[127] Souliotis V. L., Sfikakis P. P. (2015). Increased DNA double-strand breaks and enhanced apoptosis in patients with lupus nephritis. *Lupus*.

